# Novel quantitative trait loci for partial resistance to *Phytophthora sojae* in soybean PI 398841

**DOI:** 10.1007/s00122-013-2040-x

**Published:** 2013-01-25

**Authors:** Sungwoo Lee, M. A. Rouf Mian, Leah K. McHale, Hehe Wang, Asela J. Wijeratne, Clay H. Sneller, Anne E. Dorrance

**Affiliations:** 1 Department of Horticulture and Crop Science, The Ohio State University, 1680 Madison Avenue, Wooster, OH 44691 USA; 2 USDA-ARS and Department of Horticulture and Crop Science, The Ohio State University, 1680 Madison Avenue, Wooster, OH 44691 USA; 3 Department of Horticulture and Crop Science, The Ohio State University, 2021 Coffey Road, Columbus, OH 43210 USA; 4 Department of Plant Pathology, The Ohio State University, 1680 Madison Avenue, Wooster, OH 44691 USA; 5 The Molecular and Cellular Imaging Center/Ohio Agricultural Research and Development Center, 1680 Madison Avenue, Wooster, OH 44691 USA

## Abstract

Phytophthora root and stem rot caused by *Phytophthora sojae* Kaufmann and Gerdemann is one of the most severe soybean [*Glycine max* (L.) Merr] diseases in the USA. Partial resistance is as effective in managing this disease as single-gene (*Rps* gene)-mediated resistance and is more durable. The objective of this study was to identify quantitative trait loci (QTL) associated with partial resistance to *P. sojae* in PI 398841, which originated from South Korea. A population of 305 F_7:8_ recombinant inbred lines derived from a cross of OX20-8 × PI 398841 was used to evaluate partial resistance against *P. sojae* isolate C2S1 using a tray test. Composite interval mapping using a genome-wide logarithm of odd (LOD) threshold detected three QTL on chromosomes 1, 13, and 18, which individually explained 4–16 % of the phenotypic variance. Seven additional QTL, accounting for 2–3 % of phenotypic variance each, were identified using chromosome-wide LOD thresholds. Seven of the ten QTL for resistance to *P. sojae* were contributed by PI 398841. Seven QTL co-localized with known *Rps* genes and previously reported QTL for soil-borne root pathogens, isoflavone, and seed oil. Three QTL on chromosomes 3, 13, and 18 co-localized with known *Rps* genes, but PI 398841 did not exhibit an *Rps* gene-mediated resistance response following inoculation with 48 different isolates of *P. sojae*. PI 398841 is potentially a source of novel genes for improving soybean cultivars for partial resistance to *P. sojae*.

**Electronic supplementary material:**

The online version of this article (doi:10.1007/s00122-013-2040-x) contains supplementary material, which is available to authorized users.

## Introduction

Phytophthora root and stem rot is one of the most destructive diseases that suppresses soybean [*Glycine max* (L.) Merr] yield in the United States (Schmitthenner 1985). The causal agent, *Phytophthora sojae* (Kaufmann and Gerdemann), is a soilborne oomycete that germinates under wet conditions and causes seed rot or seedling damping-off of soybean at early growth stages. Under the same environmental conditions, a brown lesion begins on the lower taproot and extends up the stem in adult susceptible plants resulting in wilted and dead plants. Since it was first identified in Indiana in 1948, this disease has been found in all major soybean-growing regions around the world (Schmitthenner 1985; Anderson and Buzzell 1992; Yanchun and Chongyao 1993; Jee et al. 1998; Grau et al. 2004; Dorrance and Grünwald 2009). Annual soybean yield losses attributed to Phytophthora root and stem rot exceed $300 million in North America and $1–2 billion in worldwide production (Wrather and Koenning 2006).

Management of Phytophthora root and stem rot has primarily relied on single dominant resistance genes known as *Rps* genes. To date, 15 *Rps* genes have been identified at nine loci, and some of these have been deployed in modern soybean cultivars (Kilen et al. 1974; Mueller et al. 1978; Athow et al. 1980; Athow and Laviolette 1982; Anderson and Buzzell 1992; Buzzell and Anderson 1992; Diers et al. 1992b; Demirbas et al. 2001; Weng et al. 2001; Gordon et al. 2006; Sun et al. 2011). *Rps* gene resistance is race-specific, qualitatively inherited and confers an immune type of response following infection by *P. sojae*. However, this qualitative resistance tends to be short-lived, as *R*-genes are neutralized by adaptation of *P. sojae* populations (Schmitthenner 1985). Several mechanisms for the increasing diversity of *P. sojae* populations have been proposed, including mutation and outcrossing between different *P. sojae* strains (Förster et al. 1994). The emergence of new virulence pathotypes of *P. sojae* has limited the effectiveness of *Rps* genes deployed in commercial cultivars between 8 and 20 years (Grau et al. 2004).

Partial resistance is another type of genetic host resistance, also known as quantitative, rate-reducing, or field resistance (Tooley and Grau 1984; Walker and Schmitthenner 1984; Schmitthenner 1985). Partial resistance is quantitatively inherited, reduces the lesion development following root infection, and is effective against a wide range of *P. sojae* races (Tooley and Grau 1982; Schmitthenner 1985; Burnham et al. 2003; Mideros et al. 2007; Wang et al. 2010). Durable resistance in plants has been defined as resistance that remains effective, while it is widely used under environmental conditions favorable for disease development (Johnson 1984). For example, partial resistance in the barley (*Hordeum vulgare* L.) cultivars Minerva and Vada has been effective to barley leaf rust caused by *Puccinia hordei* Otth. for over 50 years (Parlevliet 2002), and the soybean cultivar Conrad has maintained its partial resistance in fields for over 30 years (Dorrance et al. unpublished). Soybean cultivars with high levels of partial resistance and *Rps* gene-mediated resistance were reported to be more stable for yield compared to those with either moderate or low levels of partial resistance and *Rps* gene-mediated resistance combined across a variety of environments (Dorrance et al. 2003). The combination of qualitative and quantitative resistance also prolonged effectiveness of resistance to *Leptosphaeria maculans* (Desm.) Ces. and de Not. in rapeseed (*Brassica napus* L.) (Brun et al. 2010). Moreover, partial resistance to *P. sojae* in soybean did not reduce soybean yield in the absence of disease pressure (Tooley and Grau 1984; St. Martin et al. 1994; Dorrance et al. 2003).

Several studies have mapped quantitative trait loci (QTL) for partial resistance to *P. sojae* in recombinant inbred line (RIL) populations. Two QTL for partial resistance were first mapped to chromosomes 2 and 13 in three populations, Conrad (high level of partial resistance R) × Sloan (susceptible, S), Conrad × Williams (S), and Conrad × Harosoy (S), with the alleles for partial resistance originating from Conrad (Burnham et al. 2003). Partial resistance was evaluated based on lesion length via root inoculation (tray test), and these two QTL explained a total of 42–50 % of the genotypic variance in each population (Burnham et al. 2003). Han et al. (2008) also identified QTL for partial resistance at similar locations on chromosomes 2 and 13 in a Conrad × OX760-6-1 (S) population using a different root inoculation procedure and isolates from Northeast China. Another QTL were mapped to chromosome 16 in Conrad × OX760-6-1 population based on the field-based disease incidence (Weng et al. 2007). Several QTL were detected on chromosomes 12, 13, 14, 17, 18, and 19 in a Conrad × Sloan population using a tray test or layer test. These QTL encompassed putative physiological trait genes, defense-related signaling genes, and an *R*-gene cluster (Wang et al. 2010, 2012). Four QTL were reported on chromosomes 13, 16, 18, and 20 in recombinant inbred lines (RILs) derived from an inter-specific cross between *Glycine max* V71-370 (R) and *G. soja* PI 407162 (S) (Tucker et al. 2010). Additional QTL were identified on chromosomes 2, 6, 8, 11, and 13 in a population from a cross between Conrad and Hefeng 25 (R) through field experiments conducted in multiple environments (Li et al. 2010) and on chromosomes 6, 10, and 15 in Su88-M21 (R) × Xinyixiaoheidou (S) population via a tray test (Wu et al. 2011). Recently, two more QTL on chromosomes 13 and 17 in a S99-2281(S) × PI 408105A (R) population were detected (Nguyen et al. 2012).

Very few sources of partial resistance have been used in QTL analyses and investigations into the molecular basis of partial resistance to *P. sojae,* with most studies conducted using Conrad as the source of partial resistance. Therefore, it is important to mine additional germplasm with partial resistance to *P. sojae*. More than 400 soybean accessions that have high levels of partial resistance have been identified and dozens of these accessions had higher levels of partial resistance than Conrad, including PI 398841 (Dorrance and Schmitthenner 2000). The objective of this study was to identify QTL for partial resistance to *P. sojae* using an advanced recombinant inbred (RI) population derived from the cross of OX20-8 × PI 398841, and to compare genetic locations of these QTL with previously mapped QTL.

## Materials and methods

### Plant materials and DNA extraction

A population of 305 F_7:8_ RI lines (RILs) derived from a cross of OX20-8 and PI 398841 was used for this study. Twenty-three F_1_ plants from this cross were self-fertilized to produce F_2_ seeds. The F_2_ plants were self-pollinated and each line was advanced up to the F_7_ generation by single-seed descent. OX20-8 is a breeding line developed in Ontario, Canada, which is highly susceptible (Buzzell and Anderson 1982) and PI 398841 has a high level of partial resistance to *P. sojae* (Dorrance and Schmitthenner 2000). PI 398841 was originally collected from Kwangju, South Korea.

Young leaf tissue was collected in a 2-ml tube at the V1 or V2 stage from each F_7_ plant grown in a field near the Ohio Agricultural Research and Development Center (OARDC), Wooster, OH, and flash frozen in liquid nitrogen. The frozen leaf tissue was lyophilized in a freeze drier (SP Industries Inc., Stone Ridge, NY, USA), then ground and homogenized using a Mixer Mill (Model MM301, Retsch, Hannover, Germany). DNA was extracted using a slightly modified CTAB method (Mian et al. 2008), and was dissolved in 200 μl of TE buffer (10 mM Tris–Cl, pH 7.5, 1 mM EDTA).

### Molecular marker genotyping and linkage analysis

Parental genotypes for multiple mapping populations, including OX20-8 and PI 398841, were first genotyped using the Universal Soybean Linkage Panel (USLP) 1.0 containing 1,536 SNPs at Dr. Perry Cregan’s laboratory at the United States Department of Agriculture, Agricultural Research Service, in Beltsville, MD. From this parental data, 384 SNPs were selected and organized into the Oligo Pool All (OPA) assay to genotype multiple populations using Illumina GoldenGate^®^ BeadXpress^®^ SNP genotyping (Illumina Inc., San Diego, CA, USA). In this OPA assay, 239 SNPs were polymorphic between OX20-8 and PI 398841. The SNP marker genotypes of 305 RILs were determined using the OPA according to the protocol from Illumina. The genotyping was done at the Molecular and Cellular Imaging Center (MCIC) at the OARDC.

Selected SSR markers and SSR motifs from Song et al. (2010) were used to increase genome coverage by filling large gaps in the initial SNP linkage map of the population. These 20 μl PCRs were done with 50 ng of template DNA, 1 × PCR buffer, 1.0 mM of MgCl_2_, 50 μM of each of the dNTPs, 0.1 μM of each of forward and reverse primer (IDT Inc., Coralville, IA, USA), and 1.0 U of *Taq* polymerase (GeneScript Corp., Piscataway, NJ, USA) for the final concentration. The thermal cycles began at 95 °C for 5 min, followed by 32 cycles of denaturing at 95 °C for 30 s, annealing at 48–61 °C (according to the optimum temperature for the primer pair used) for 30 s, and extension at 72 °C for 45 s. Additional 10 min of extension at 72 °C followed at the end of the last cycle. The PCR product was resolved on a 4 % high-resolution agarose gel (Research Products International Corp., Mt. Prospect, IL, USA) by gel electrophoresis.

The genetic map was constructed with JoinMap^®^ 4 (Van Ooijen 2006) using the Kosambi mapping function. Linkage was determined at the logarithm of odd (LOD) threshold of 3.0 with a maximum map distance of 50 centiMorgan (cM). The order of markers in linkage groups was compared with the Consensus Map 4.0 (Hyten et al. 2010).

### Pathogenicity test of *P. sojae* isolates

The hypocotyl inoculation technique (Dorrance et al. 2008) was used to test pathogenicity of *P. sojae* isolates to identify isolates that were virulent or avirulent to OX20-8 and PI 398841. In brief, 10–15 seeds were placed on a germination paper, and the paper was then rolled and placed in a plastic bucket under dark conditions at 25 °C. Seven-day-old etiolated seedlings were inoculated with *P. sojae* inoculum slurry from 7-day-old cultures grown on lima bean agar (15 g agar/l). The papers were rolled and kept in a plastic bucket in darkness. Plants that have an *R* gene developed a hypersensitive reaction around the inoculation site, while susceptible plants developed an expanding brown lesion in 3–5 days. Plants were scored based on the percentage of susceptible interactions 7 days after inoculation as follows: <25 % plants killed as resistant; 25–75 % plants killed as intermediate; and >75 % plants killed as susceptible.

Those which induce the susceptible response on both parents are appropriate isolates for testing partial resistance, because the expression of partial resistance may be masked by the hypersensitive response caused by *Rps* genes if either parent has a *Rps* gene interacting with *Avr* genes of the same isolates. Alternatively, isolates that induced an *Rps* gene-mediated resistance response following inoculation of PI 398841, but virulent to OX20-8, could be used to detect and map *Rps* genes that may exist in PI 398841.

### Partial resistance evaluation

Lesion lengths following *P. sojae* inoculation on roots were measured to evaluate levels of partial resistance among the RILs using a tray test assay which was previously published (Burnham et al. 2003; Tucker et al. 2010; Wang et al. 2010). Briefly, ten 7-day-old seedlings from each RIL were placed on a tray and a 1-cm wound was made on the tap root 20 mm below the crown with a scalpel. An agar-mycelial slurry from a 7-day-old culture of *P. sojae* isolate C2S1 (vir 1a, 1b, 1c, 1 k, 2, 3a, 3b, 3c, 4, 5, 6, and 7) was placed over the wound. After 7 days, lesion lengths were measured from the inoculation site to the edge of the lesion margin. The isolate C2S1 was one of the isolates which has a susceptible response to both parents, and the lesion length was significantly different between parents in a preliminary tray test compared to the other isolates that induced susceptible responses (data not shown).

A total of 305 RILs were separated into two sets consisting of 158 and 147 RILs. The first set was evaluated in 2010 (January to March) and the second in 2011 (January to March), respectively. Each set was divided into two incomplete blocks. An incomplete block consisted of six buckets, each of which included 16 RILs, two parents, and two checks, ‘Conrad’ and ‘Sloan’. Conrad and Sloan represent high level of partial resistance and moderate susceptibility, respectively (Burnham et al. 2003). There were three replications.

The data from both sets of RILs from the each experiment were combined and the mean lesion length of ten seedlings from each RIL was analyzed to obtain the best linear unbiased predictor (BLUP) using the PROC MIXED procedure in SAS (SAS 9.1, SAS Institute Inc., Cary, NC, USA) (Stroup 1989). The model was *Y*_*ijkl*_ = *μ* + *S*_*i*_ + *R*(*S*)_*ij*_ + *I*(RS)_*ijk*_ + *B*(IRS)_*ijkl*_ + *C*_*m*_ + *G*(*C*)_*mn*_ + *ε*_*ijklmn*_, where *μ* is the overall mean, *S*_*i*_ is the effect of *i*th set, *R*_*j*_ is the effect of *j*th replication in *i*th set, *I*(SR)_*ijk*_ is the effect of *k*th incomplete block in *j*th replication in *i*th set, *B*(SRI)_*ijkl*_ is the effect of *l*th bucket in *k*th incomplete block and *j*th replication in *i*th set, *C*_*m*_ is the effect of *m*th class of entry (*l* = 1, 2, 3, 4, and 5 for, OX20-8, PI 398841, Conrad, Sloan, and RIL, respectively), *G*(*C*)_*mn*_ is the effect of *n*th genotype within class for recombinant inbred lines only (genotypic variance, *σ*_G_^2^), *ε*_*ijklm*_ is the experimental error (*σ* ^2^). Class of entry was assumed to be a fixed effect and all other terms random effects. Variance components were estimated using the restricted maximum likelihood (REML) method (Patterson and Thompson 1971). The heritability, on a line-mean basis, was calculated as *σ*_G_^2^/(*σ*_G_^2^ + *σ* ^2^/*r*), where *r* is the number of replications per RIL.

### QTL analysis, detection of epistasis and confirmation of QTL effects

Kruskal–Wallis analysis and interval mapping (IM) were initially performed to identify potential QTL, followed by composite interval mapping (CIM) using multiple-QTL method (MQM) with cofactors in MapQTL^®^ 5 (Van Ooijen 2004). Walking speed was set to 1 cM for both IM and CIM. The genome-wide and chromosome-wide LOD threshold was determined by conducting a 1,000-permutation test at *α* = 0.05 (Churchill and Doerge 1994). Multiple regression analysis was conducted using PROC REG in SAS with the nearest markers to the QTL identified by CIM to calculate the total phenotypic variance (*R* ^2^). The LOD plots for the chromosomes on which a significant QTL were identified were graphically presented using the MapChart 2.2 software (Voorrips 2002). To test the interaction between identified QTL, genotypic data of the markers nearest to each QTL were analyzed by PROC GLM in SAS (SAS 9.1, SAS Institute Inc., Cary, NC, USA). The statistical model was *Y*_*ij*_ = *μ* + *M*1_*i*_ + *M*2_*j*_ + (*M*1_*i*_ × *M*2_*j*_) + *ε*_*ij*_, where *Y*_*ij*_ is the phenotypic values (BLUP) for entries in the *i*th and *j*th marker classes for *M*1 and *M*2, *μ* is the overall mean, *M*1_*i*_ is the effect of the *i*th marker class for *M*1, *M*2_*j*_ is the effect of the *j*th marker class for *M*2, *ε*_*ij*_ is the residual error. The appropriate *F* test for epistasis between molecular markers was *F* = (MS*M*1_*i*_ × *M*2_*j*_)/(MS*ε*_*ij*_).

Using the QTL identified with a genome-wide LOD threshold, the additivity of accumulated QTL effects on levels of partial resistance was verified. The cumulative additive effects (CAEs) of RILs provided by the alleles for the QTL were obtained based on the genotypes of the nearest markers to the QTL. The estimated additive effect of individual QTL was given to RIL that were homozygous for the resistance allele for the nearest marker to the corresponding QTL, while an effect of zero was given to those who were homozygous for the recessive alleles for the same marker. Then, a CAE of individual RIL was obtained by adding all given additive effects. Statistical significance of the regression of BLUP values on the CAEs was tested to confirm the trend of levels of partial resistance in relations with the cumulative additive effects using PROC REG in SAS.

## Results

### Partial resistance evaluation and statistical analysis

The mean lesion length was significantly different among the two parental lines and the two checks in all buckets (data not shown), indicating that PI 398841 had high levels of partial resistance to *P. sojae*. The mean lesion lengths averaged over all buckets from both sets were 50.1, 41.3, 29.6, and 25.5 mm for OX20-8, Sloan, Conrad, and PI 398841, respectively; the four means were separated from one another by Fisher’s least square difference (PROC GLM, *P* < 0.0001). PI 398841, the parent with partial resistance, had significantly shorter lesions than Conrad, which has been known to have high levels of partial resistance to *P. sojae*. However OX20-8, the susceptible parent, had significantly longer lesions than Sloan, which is highly susceptible. The mean lesion length of the individual RILs ranged from 12.6 to 67.6 mm, and the overall mean of all of the RILs was 39.1 mm.

**Fig. 1:**
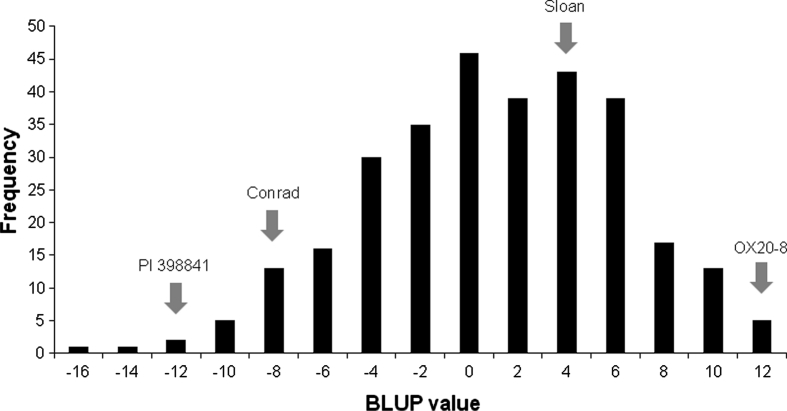
Frequency distribution of BLUP values for mean lesion length of RILs derived from OX20-8 × PI 398841 population. Estimates of two parents and two checks are indicated by *arrows*. A lower BLUP value means a higher level of partial resistance to *P. sojae*. OX20-8, the susceptible parent; PI 398841, the resistant parent; Conrad, the check for high level of partial resistance; Sloan, the check for low level of partial resistance

The BLUP values were calculated from the mean lesion length, and the frequency of BLUP values was normally distributed in this population (Fig. 1). Lower BLUP values indicate higher levels of partial resistance. The BLUP values estimated from the mixed model analysis were −13.6 and 11.1 for PI 398841 and OX20-8, respectively. The BLUP values of the checks were also separated with −9.4 and 2.3 for Conrad and Sloan, respectively. Two RILs had significantly lower BLUP values than PI 398841, the resistance parent, which indicates potential transgressive segregation. The mean lesion lengths of these two RILs were shorter than those of PI 398841 in each of the experiments across all replications, with one missing value for one of the RILs (data not shown). One-tailed paired *t* test with each RIL and PI 398841 in the same experimental units indicated one transgressive segregant (*P* < 0.05). Broad-sense heritability of mean lesion length was moderately high (0.77).

### Linkage analysis

Two hundred and twenty SNP genotypes of 305 RILs were determined with the OPA containing 239 polymorphic SNPs for this population. Nineteen SNPs were not polymorphic nor scorable. Of these 220 SNP markers, 214 were mapped to construct the initial genetic map of the population. To increase the genome coverage by closing gaps found in the initial map, 59 SSR markers were added to the linkage map. A total of 214 SNPs and 59 SSRs were finally integrated into the genetic map of the OX20-8 × PI 398841 population for QTL analysis (Supplementary Fig 1). A total of 37 linkage fragments were generated, spanning 2,160 cM. This OX20-8 × PI 398841 genetic map corresponds to approximately 1,537 cM of the soybean Consensus Map 4.0, with 69 % coverage of the genome according to the genetic position (cM) of molecular markers shared between the two maps. Most of the shared markers were ordered in agreement with the Consensus Map 4.0 (Hyten et al. 2010) and only closely linked markers had an order which differed from that in the Consensus Map 4.0.

### QTL identification by the genome-wide LOD threshold

**Fig. 2:**
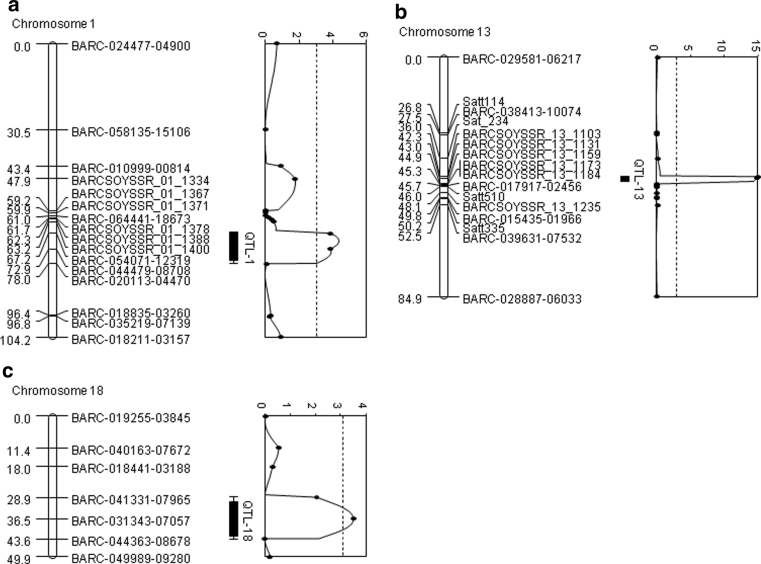
Graphical presentations of three quantitative trait loci for partial resistance to *P. sojae* identified using the genome-wide LOD threshold in the F_7:8_ RIL population derived from a cross of OX20-8 × PI 398841. Genetic distance (cM) and marker names are shown to the left and right of chromosomes, respectively. The LOD plots to the right of chromosomes indicate the most likely position of QTL conferring partial resistance to *P. sojae*. The *hatched lines* on the LOD plots indicate the genome-wide LOD threshold for CIM of 3.1. The 1- and 2-LOD intervals are displayed as *black bars* and *solid lines*, respectively

Three QTL associated with partial resistance to *P. sojae* were identified on chromosomes 1, 13, and 18 by composite interval mapping (Table 1; Fig. 2). The major QTL closely linked to BARCSOYSSR_13_1103 on the chromosome 13 (QTL-13) accounted for 16.1 % of the phenotypic variance (Table 1; Fig. 2). Two minor QTL adjacent to BARC-044479-08708 on chromosome 1 (QTL-1) and BARC-031343-07057 on chromosome 18 (QTL-18) explained 4.6 and 3.6 % of the phenotypic variance, respectively (Table 1; Fig. 2). The resistance alleles for all the QTL were contributed by PI 398841 as indicated by the additive effects of negative values (Table 1). A total of 28.8 % of the phenotypic variance were explained by the three QTL. Two-way ANOVA did not detect any epistatic allelic interactions between markers that were significant in Kruskal–Wallis analysis (data not shown).

**Table 1:** Identification of three quantitative trait loci associated with partial resistance to *P. sojae* in the OX20-8 × PI 398841 population using the genome-wide LOD threshold

Chr.^a^	Position (cM)^b^	Nearest marker	LOD^c^	*R* ^2^ (%)^d^	Additive effect (contributor)^e^
1	71	BARC-044479-08708	4.4	4.6	−1.26 (PI 398841)
13	51	BARCSOYSSR_13_1103	14.8	16.1	−2.12 (PI 398841)
18	91	BARC-031343-07057	3.4	3.6	−1.02 (PI 398841)

^a^Chromosome numbers

^b^Position (cM) on Consensus Map 4.0 (Hyten et al. 2010). Since BARCSOYSSR_13_1103 was not integrated on the Consensus Map 4.0, an approximate position was denoted based on the adjacent marker, Satt334, on the Williams82 genome assembly Glyma1.01

^c^A genome-wide LOD threshold was 3.1 at 95 % of confidence level by a 1,000-permutation test

^d^Phenotypic variance explained by the QTL

^e^The negative additive effects indicate that PI 398841 contributes favorable alleles for the QTL

### Effects of combining the resistant alleles for the QTL

**Fig. 3:**
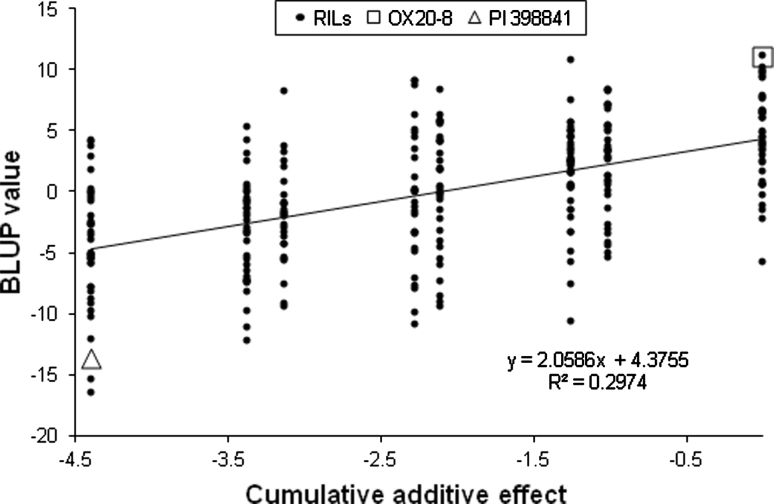
Regression of the BLUP values on cumulative additive effects of pyramiding resistance alleles for QTL conferring partial resistance to *P. sojae* identified in this study. The accumulative additive effects were calculated based on three QTL, QTL-1, -13, and -18. *X*-axis indicates the sum of additive effects of combined resistance alleles for QTL in each RIL and two parental lines and *Y*-axis indicates BLUP values of parents and RILs. Lower BLUP values mean higher levels of resistance. The additive effects of resistance alleles for the three QTL identified in this study have negative values. The equation and *R* ^2^ of the regression line are shown on the scatter plots. The BLUP values of OX20-8, PI 398841, and RILs are plotted as *square*, *triangle*, and *black circles*

Eight different CAEs were obtained from the eight allelic combinations for the three QTL, which ranged −4.4 to 0. RILs that have homozygous PI 398841 alleles for all three QTL were given −4.4, and those with homozygous OX20-8 alleles for the three were given 0, since the resistance allele was contributed by PI 398841 for all three QTL. The BLUP values of RILs were visualized on their own CAEs (Fig. 3). RILs with the same CAE composed each vertical line of dots. The regression coefficient of 2.1 was significantly different from zero (*P* < 0.0001), indicating that levels of partial resistance to *P. sojae* decreased as the susceptible alleles were accumulated in a RIL. The *R* ^2^ value of the regression line was 0.29, which was somewhat higher than the sum of *R* ^2^ (%) values of three QTL. BLUP values of RILs were dispersed evenly on the same CAE since RILs were segregating for much more than three QTL used for this regression analysis and the three QTL accounted for the limited proportion of phenotypic variance, which implied that there was still a large proportion of phenotypic variance that was not explained.

### Indication of additional QTL by chromosome-wide LOD thresholds

**Fig. 4:**
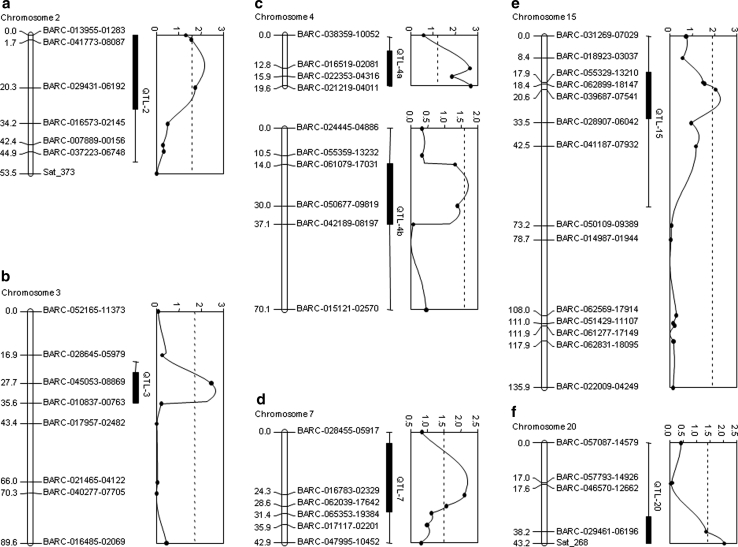
Graphical presentations of seven additional quantitative trait loci for partial resistance to *P. sojae* identified using chromosome-wide LOD thresholds in the F_7:8_ RIL population derived from a cross of OX20-8 × PI 398841. Genetic distance (cM) and marker names are shown to the left and right of chromosomes, respectively. The LOD plots to the right of chromosomes indicate the most likely position of QTL conferring partial resistance to *P. sojae*. The *hatched lines* on the LOD plots indicate the chromosome-wide LOD threshold for CIM. The 1- and 2-LOD intervals are displayed as *black bars* and *solid lines*, respectively

Chromosome-wide LOD thresholds (*α* = 0.05) allowed the detection of seven additional putative QTL (Table 2; Fig. 4). Four putative QTL were mapped near BARC-029431-01692, BARC-045053-08869, BARC-016783-02329, and Sat_268 on chromosomes 2, 3, 7, and 20 (QTL −2, −3, −7, and −20), respectively. These QTL individually explained 1.9–2.8 % of phenotypic variance and the resistance alleles were contributed by PI 398841. In concordance with the transgressive segregation observed (Fig. 1), susceptible genotype OX20-8 also contributed three putative QTL. They were identified near BARC-021219-04011 and BARC050677-09819 on chromosomes 4 and BARC-039687-07541 on chromosomes 15 (QTL-4a, QTL-4b, and QTL-15), which individually accounted for 1.9-2.6 % of phenotypic variance. The QTL-4a and -4b were located on two separate linkage groups from chromosome 4.

**Table 2:** Seven putative quantitative trait loci for partial resistance to *P. sojae* in the OX20-8 × PI 398841 population using chromosome-wide LOD thresholds

Chr.^a^	Position (cM)^b^	Nearest marker	LOD	LOD threshold^c^	*R* ^2^ (%)^d^	Additive effect (contributor)^e^
2	20	BARC-029431-01692	2.2	1.6	2.3	−0.88 (PI 398841)
3	28	BARC-045053-08869	2.6	1.7	2.8	−0.92 (PI398841)
4	20	BARC-021219-04011	2.6	1.2	2.5	0.85 (OX20-8)
4	64	BARC-050677-09819	1.7	1.6	1.9	0.78 (OX20-8)
7	50	BARC-016783-02329	2.2	1.5	2.4	−0.87 (PI 398841)
15	19	BARC-039687-07541	2.3	1.9	2.6	0.92 (OX20-8)
20	48	Sat_268	2.0	1.4	1.9	−0.74 (PI 398841)

^a^Chromosome numbers

^b^Position (cM) on Consensus Map 4.0 (Hyten et al. 2010)

^c^Chromosome-wide LOD thresholds at *α* = 0.05 were calculated by a 1,000-permutation test

^d^Phenotypic variance explained by the QTL

^e^The negative additive effects indicate that PI 398841 contributes favorable alleles for the QTL

### Presence of *Rps* genes

Forty-eight isolates of *P. sojae* were screened on parental genotypes and 15 differentials via hypocotyl inoculation technique to identify isolates that had a qualitative resistance response in PI 398841 which indicates the presence of *Rps* genes. Although a diverse collection of *P. sojae* pathotypes was used, PI 398841 did not exhibit *Rps* gene-mediated resistance to any of the isolates used in this study. PI 398841 had susceptible responses to 42 isolates of *P. sojae* and intermediate responses to six of the 48 isolates used to identify *Rps* genes in this study (Supplementary Table 1). OX20-8, which has *Rps1a*, had a susceptible, intermediate and resistant response following inoculation to 42, 3 and 3 isolates of *P. sojae*, respectively (Supplementary Table 1).

## Discussion

In this study, a large number of highly homozygous RILs derived from a cross of OX20-8 and PI 398841 were evaluated via tray tests with three replications for resistance to *P. sojae*. A high broad-sense heritability was estimated for partial resistance to *P. sojae* in this population (*H* = 0.77), which is in agreement with several previous studies which had heritability estimates ranging from 0.59 to 0.92 (Burnham et al. 2003; Wang et al. 2010; Tucker et al. 2010; Wu et al. 2011). The moderate to high heritability indicates that partial resistance to *P. sojae* would be inherited stably, and could, therefore, be selected effectively in a breeding program. Moreover, PI 398841 has higher levels of partial resistance than Conrad in highly replicated experiments with the data collected from 12 independent tests (4 subsets × 3 replications). Therefore, PI 398841 is a potential source for high levels of partial resistance to *P. sojae*.

The QTL-13 contributed a major effect for partial resistance to *P. sojae* (Fig. 2). Based on the position of the closest marker BARCSOYSSR_13_1103, the QTL-13 was located near the markers associated with partial resistance to *P. sojae* previously reported in populations derived from a cross of V71-370 (R) × PI 407162 (S) (Tucker et al. 2010) and Conrad (R) × Sloan (S) (Wang et al. 2010). The QTL identified by Tucker et al. (2010) were flanked by Satt114 and Satt510, and the QTL-13 from the present study is within this interval. The QTL from Conrad × Sloan were located between Satt510 and Sct_033 (Wang et al. 2010), an interval outside of the one containing QTL-13, but partially overlapping with the QTL identified by Tucker et al. (2010) (Fig. 2). QTL-13 had a much higher LOD score (14.8) and explained a greater portion of the phenotypic variance (16.1 %) than QTL reported from at this location in two previous studies (Tucker et al. 2010; Wang et al. 2010). This genomic region is well known as an *R* gene and defense gene rich region. Two closely linked *P. sojae* resistance genes, *Rps3* and *Rps8*, were mapped in this region (Gordon et al. 2006). In addition, *Rpg1* for resistance to bacterial blight caused by *Pseudomonas savastanoi* pv. *glycinea*, *Rsv1* for resistance to *soybean mosaic virus*, *Rpv1* for resistance to *peanut mottle virus*, *Rag2* for resistance to soybean aphid (*Aphis glycines* Matsumura), and a QTL for resistance to *Sclerotinia sclerotiorum* (Lib.) de Bary were mapped in this *R* gene cluster (Arahana et al. 2001; Ashfield et al. 1998; Gore et al. 2002; Jeong et al. 2001; Mian et al. 2008). Weak forms of *R* genes have been considered as a potential mechanism for expressing partial resistance, and quantitative disease resistance loci that co-localized with *R* genes have been reported in many crop species (Li et al. 1999, 2006; Poland et al. 2009; St. Clair 2010).

Two novel but minor QTL were also identified on chromosomes 1 and 18 (Table 1; Fig. 2a, c). QTL-1 is a novel locus associated with partial resistance to *P. sojae* and no *Rps* gene has been mapped to date on chromosome 1. The QTL for seed weight (Sd wt 15-2 and Sd wt 18-1.1) and oil (oil 24-21) have also been reported near this region (Hyten et al. 2004; Panthee et al. 2005; Qi et al. 2011). Coincidence of QTL for *P. sojae* partial resistance with QTL for seed yield and oil was described in an earlier study (Wu et al. 2011). Further studies are needed to explain any relationship between *P. sojae* partial resistance and seed quality-related traits. The QTL-18 is another novel QTL for partial resistance to *P. sojae*; though another QTL for partial resistance to *P. sojae* were mapped to a position 10 cM “below” and were consistently identified against multiple *P. sojae* isolates via tray tests and layer tests, explaining 11–23 % of phenotypic variance (Wang et al. 2012). The QTL-18 region was also located near *Rps4*, *5*, and *6* (Demirbas et al. 2001). *Rps4* and *6* have been mapped adjacent to Satt472 or Satt191 (Demirbas et al. 2001). The nearest marker BARC-031343-07057 to QTL-18 was located only 1 or 5 cM “above” Satt191 or Satt472, based on marker positions on the Consensus Map 4.0 (Hyten et al. 2010). In addition, a QTL for resistance to soybean cyst nematode (SCN), *Heterodera glycines* Ichinohe, was identified between Satt612 and Satt191 near the QTL-18 (Vuong et al. 2010).

Since the majority of partial resistance QTL identified in previous studies had small-effects and their significance was low, some minor QTL may be overlooked due to statistical insignificance for the given threshold. A genome-wide LOD threshold (i.e., experiment-wise error rate) at *α* = 0.05 is generally accepted for a standard to determine significant linkage in QTL mapping. Lander and Kruglyak (1995) proposed “suggestive linkage”, which denoted weak associations between genotype and phenotype with no statistical significance at the experiment-wise error rate. A chromosome-wide LOD threshold was proposed for determining “suggested linkage” as the analysis of the markers on a single chromosome can be considered as a separated experiment, and LOD thresholds are dependent on chromosome map lengths (Van Ooijen 1999). Chromosome-wide LOD thresholds (*α* = 0.05) allow for the identification of QTL which may contribute a minor proportion of the overall effect; consequently, type I errors may occur at a higher rate. The main advantage is enabling the identification of potential QTL with low levels of significance, but which may be real, over multiple experiments. Recurrent emergence of particular loci could allow for the assembly of complex genetic networks for complex traits such as partial resistance to *P. sojae*. Thus, this study also reported putative QTL based on significance at chromosome-wide LOD thresholds.

Seven putative QTL with relatively low LOD values (1.7–2.6) were identified based on chromosome-wide LOD thresholds (Table 2; Fig. 4). Near the QTL-2, one QTL for oil content was reported (Zhang et al. 2004). The QTL-3 overlapped to loci closely linked to *Rps1* and *7* (Weng et al. 2001). QTL associated with isoflavone have been reported near the QTL-7 in previous studies (Primomo et al. 2005; Zeng et al. 2009). This phenomenon has also been described in Wu et al. (2011). In addition, QTL for sudden death syndrome (SDS) caused by *Fusarium virguliforme* as well as isoflavone had been mapped near the QTL-20 (Iqbal et al. 2001; Zeng et al. 2009). Isoflavone accumulation in soybean roots is thought to play an important role in resistance for soybean-*P. sojae* and -*F. virguliforme* interactions (Lozovaya et al. 2004; Subramanian et al. 2005). Interestingly, QTL where resistance alleles were contributed by the susceptible parent also co-localized with or neighbored QTL for soil-borne diseases. Two loci for resistance to SCN (SCN21-1 and 18-3) were previously reported within QTL-4a interval and QTL-4b was mapped within 5 cM of a QTL for resistance to SDS (SDS9-3) (Yue et al. 2001; Njiti and Lightfoot 2006). The QTL-15 region also overlapped QTL conferring resistance to *P. sojae*, *S. sclerotiorum*, and *H. glycines* (Qiu et al. 1999; Arahana et al. 2001; Wu et al. 2011). In addition, a few studies had identified the region within the QTL-15 interval as highly associated with seed oil (Diers et al. 1992a; Lee et al. 1996). The QTL for partial resistance to *P. sojae* identified in this study are coincident with QTL for isoflavone, oil, or resistance to other soil-borne pathogens. This co-localization indicates that a portion of the ten QTL may include genes involved in conserved roles in mediating resistance to various soil-borne pathogens and potentially the genes at these loci may have pleiotropic effects. Additional mapping of more sources of resistances for these traits as well as functional studies of the genes associated with these traits will be necessary.

In previous studies, most of the QTL associated with partial resistance to *P. sojae* have been mapped in regions distinct from those known to contain *Rps* genes. In addition to the *Rps3* and *8* cluster, the *Rps1* and *7* cluster as well as the *Rps4*, *5*, and *6* cluster were first reported to be associated with partial resistance to *P. sojae* in PI 398841. One of the hypotheses of quantitative resistance summarized by Poland et al. (2009) is that defeated or alternative forms of *R*-genes may contribute to the expression of quantitative disease resistance. The mapping of *Rps* genes in the OX20-8 × PI 398841 population would provide further evidence for or against this hypothesis. To find isolates causing a resistant reaction in PI 398841, but a susceptible reaction in OX20-8, 48 isolates of *P. sojae* were used to inoculate parents and 15 differentials via the hypocotyl inoculation technique. PI 398841, however, did not exhibit a resistant response to any of the isolates with diverse pathotypes, including OH 1, which has one virulence gene to *Rps7* (Supplementary Table 1). Some possible interpretations of this result are that (1) *Rps* genes may exist in PI 398841 that may have incomplete expression similar to *Rps2*; (2) PI 398841 may have alleles of *Rps* genes that were not effective towards the pathotypes tested in this study; or (3) there are no *Rps* genes present in PI 398841.

There was a considerable gap between the phenotypic variance (0.41) when all of the QTL were considered and the estimated heritability (0.77). Possible explanations for the remainder of genetic components include the following: (1) some QTL are too small to detect via designated experiments, (2) unknown QTL may be located on the regions not covered in the genetic map used in the study, (3) multi-gene interactions were unable to be significantly detected in this study, or (4) the heritability may be somewhat overestimated. Recent studies noted the importance of epistatic QTL, which are interacting loci without significant individual effects on the given trait, in highly complex traits such as fatty acid composition and drought tolerance (Ravi et al. 2011; Yang et al. 2010). Though no significant QTL × QTL interaction was detected by two-way ANOVA, genome-wide scans for epistatic effects could provide more information about genetic network of partial resistance to *P. sojae*.

This study reported a total of ten QTL associated with partial resistance to *P. sojae* from a OX20-8 × PI 398841 population. Seven QTL of the ten detected in this study are reported for the first time here. In comparison with previously reported QTL, the majority of the ten QTL were located near *R* gene rich regions, including *Rps* genes, or regions for resistance to soil-borne fungal pathogens. Three were mapped near QTL regions associated with oil or isoflavone. Further studies will confirm the QTL in the next generation and with near-isogenic lines. Detection of any isolate-specificity of QTL will be especially important due to the ability of pathogen populations to rapidly evolve. Thus, the comparison of QTL identified against multiple isolates in multiple populations sharing a common parent will be critical to identify durable QTL for partial resistance.

## Electronic supplementary material

Below is the link to the electronic supplementary material.

Supplementary material 1 A genetic map of OX20-8 × PI 398841 population (PDF 74 kb)

Supplementary material 2 The response of OX20-8, PI 398841, and fifteen differentials following inoculations with forty-eight isolates of *Phytophthora sojae* (DOCX 29 kb)
