# Supplementary material for: Novel quantitative trait loci for partial resistance to Phytophthora sojae in soybean PI 398841
Source: Theor Appl Genet. 2013 Jan 25;126(4):1121–32. doi: 10.1007/s00122-013-2040-x (PMC3607739; doi:10.1007/s00122-013-2040-x)
Supplement: Supplementary file 1 — Supplementary material 1 A genetic map of OX20-8 × PI 398841 population (PDF 74 kb) [file 122_2013_2040_MOESM1_ESM.pdf]

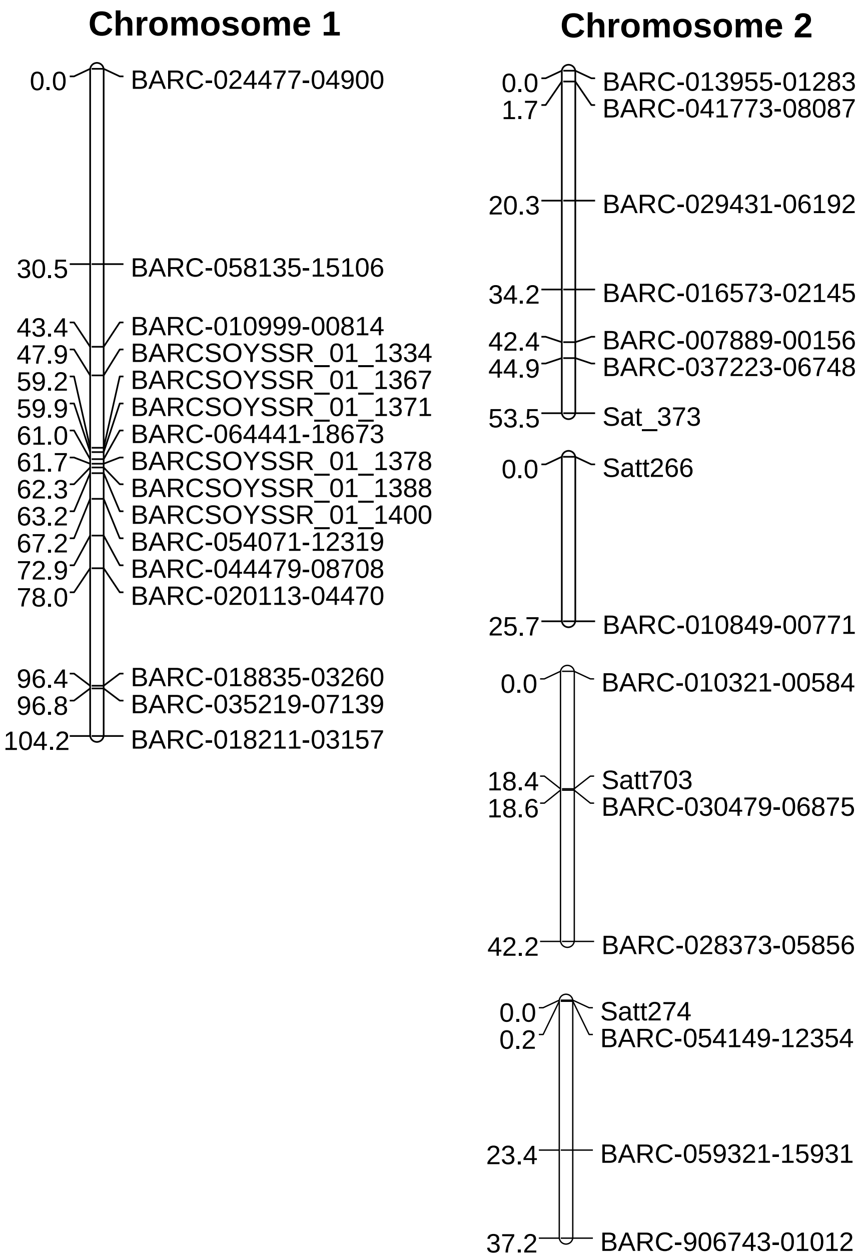

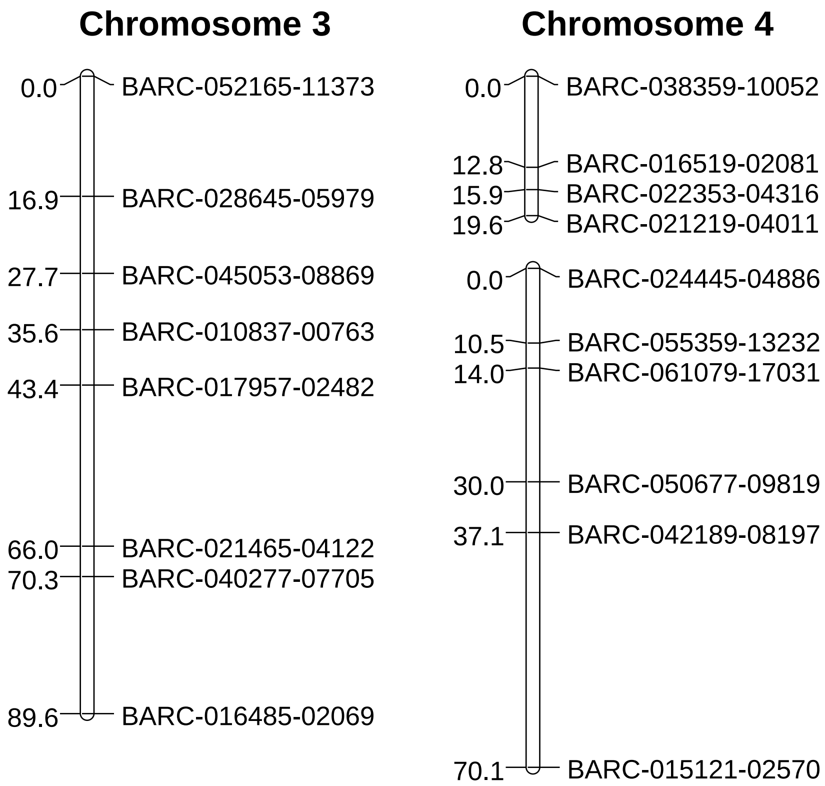

Electronic Supplementary Fig 1
A genetic map of OX20-8 × PI 398841 population.

Title: Novel quantitative trait loci for partial resistance to *Phytophthora sojae* in soybean PI 398841.

Journal: Theoretical and Applied Genetics.

Authors: Sungwoo Lee, M. A. Rouf Mian, Leah K. McHale, Hehe Wang, Asela J. Wijeratne, Clay H. Sneller, Anne E. Dorrance

Corresponding author: Anne E. Dorrance (dorrance.1@osu.edu), The Ohio State University,

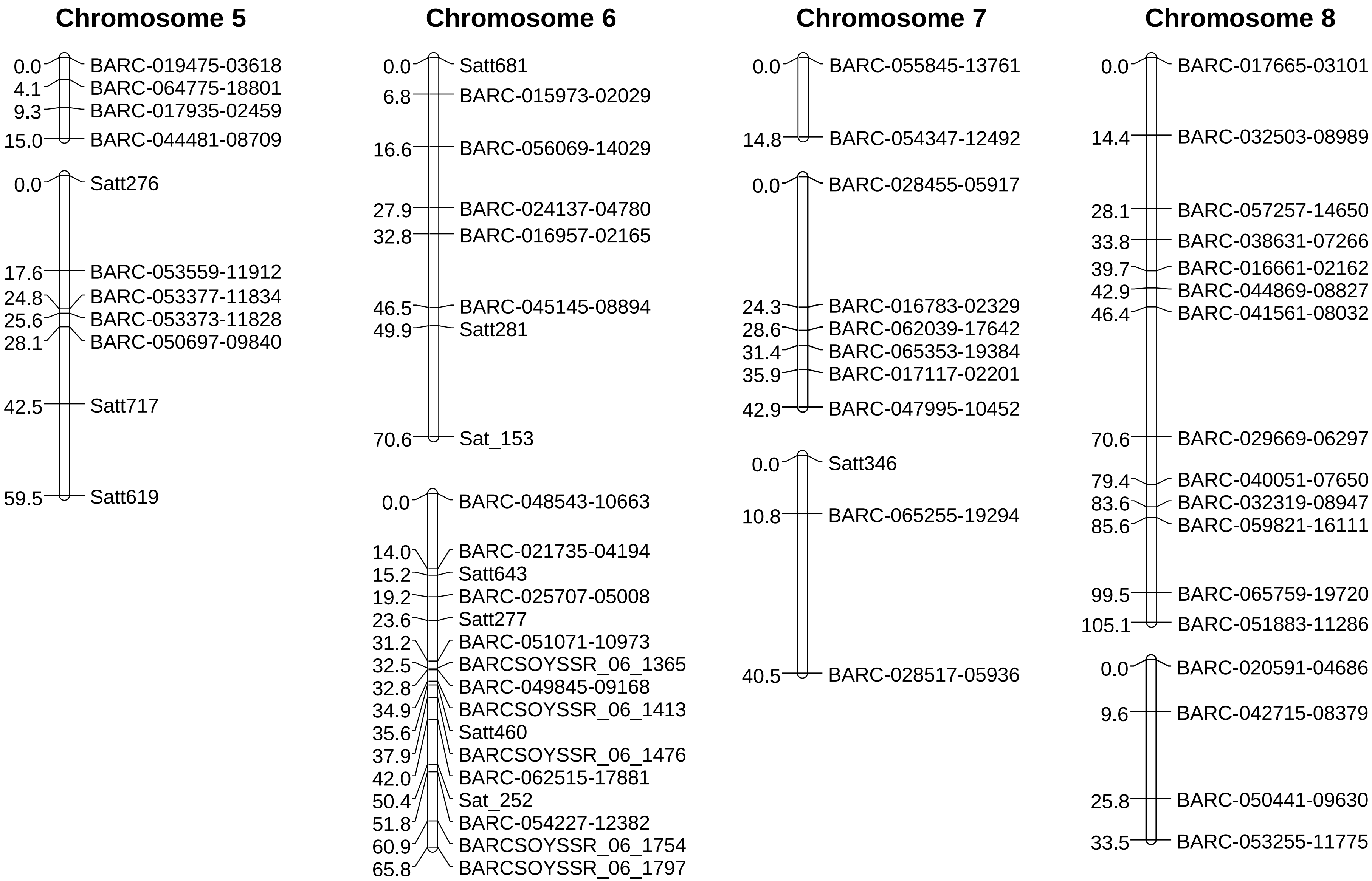
Chromosome 5
0.0 BARC-019475-03618
4.1 BARC-064775-18801
9.3 BARC-017935-02459
15.0 BARC-044481-08709
0.0 Satt276
17.6 BARC-053559-11912
24.8 BARC-053377-11834
25.6 BARC-053373-11828
28.1 BARC-050697-09840
42.5 Satt717
59.5 Satt619
Chromosome 6
0.0 Satt681
6.8 BARC-015973-02029
16.6 BARC-056069-14029
27.9 BARC-024137-04780
32.8 BARC-016957-02165
46.5 BARC-045145-08894
49.9 Satt281
70.6 Sat_153
0.0 BARC-048543-10663
14.0 BARC-021735-04194
15.2 Satt643
19.2 BARC-025707-05008
23.6 Satt277
31.2 BARC-051071-10973
32.5 BARCSOYSSR_06_1365
32.8 BARC-049845-09168
34.9 BARCSOYSSR_06_1413
35.6 Satt460
37.9 BARCSOYSSR_06_1476
42.0 BARC-062515-17881
50.4 Sat_252
51.8 BARC-054227-12382
60.9 BARCSOYSSR_06_1754
65.8 BARCSOYSSR_06_1797
Chromosome 7
0.0 BARC-055845-13761
14.8 BARC-054347-12492
0.0 BARC-028455-05917
24.3 BARC-016783-02329
28.6 BARC-062039-17642
31.4 BARC-065353-19384
35.9 BARC-017117-02201
42.9 BARC-047995-10452
0.0 Satt346
10.8 BARC-065255-19294
40.5 BARC-028517-05936
Chromosome 8
0.0 BARC-017665-03101
14.4 BARC-032503-08989
28.1 BARC-057257-14650
33.8 BARC-038631-07266
39.7 BARC-016661-02162
42.9 BARC-044869-08827
46.4 BARC-041561-08032
70.6 BARC-029669-06297
79.4 BARC-040051-07650
83.6 BARC-032319-08947
85.6 BARC-059821-16111
99.5 BARC-065759-19720
105.1 BARC-051883-11286
0.0 BARC-020591-04686
9.6 BARC-042715-08379
25.8 BARC-050441-09630
33.5 BARC-053255-11775

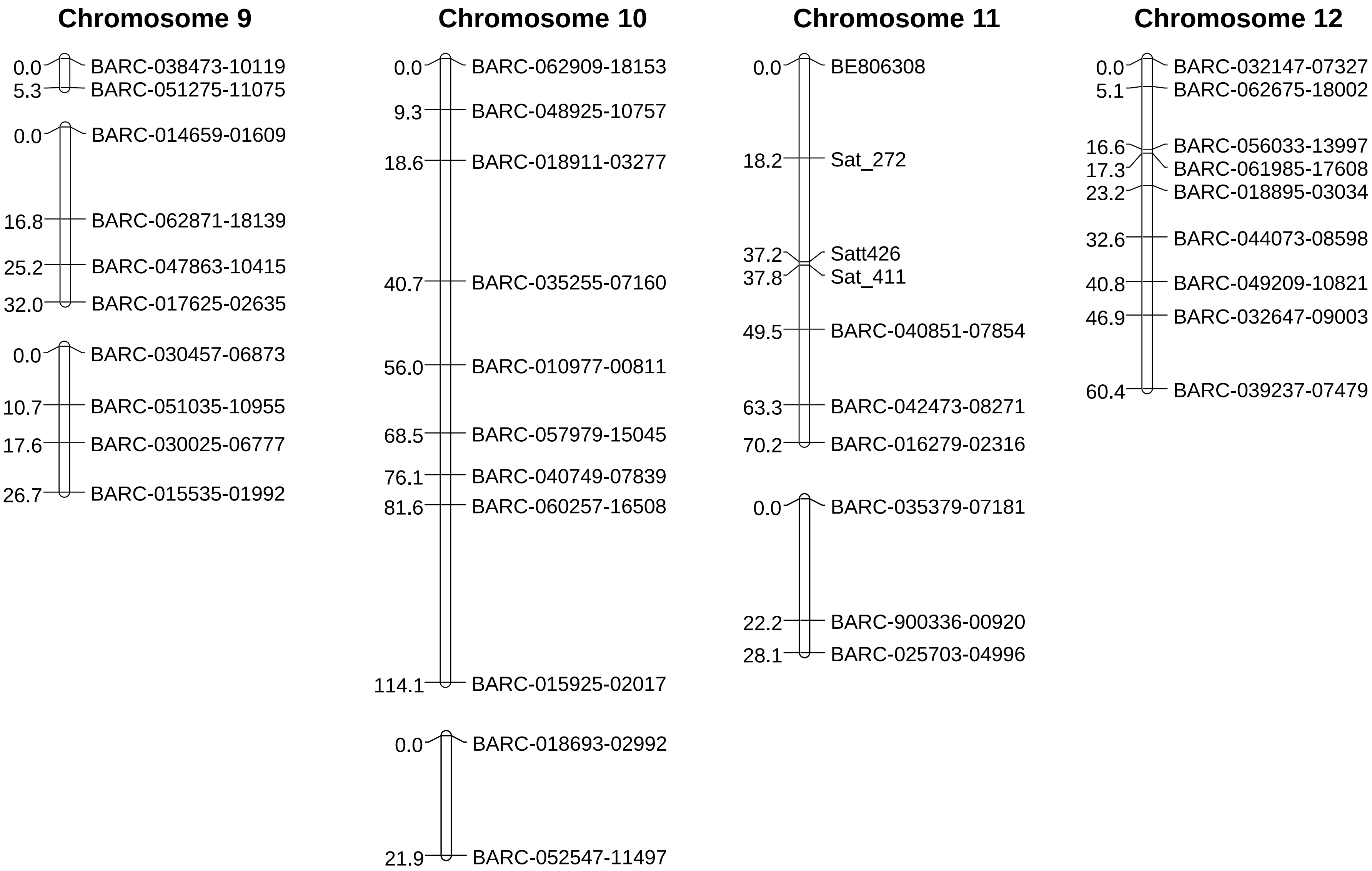
Chromosome 9
0.0 BARC-038473-10119
5.3 BARC-051275-11075
0.0 BARC-014659-01609
16.8 BARC-062871-18139
25.2 BARC-047863-10415
32.0 BARC-017625-02635
0.0 BARC-030457-06873
10.7 BARC-051035-10955
17.6 BARC-030025-06777
26.7 BARC-015535-01992
Chromosome 10
0.0 BARC-062909-18153
9.3 BARC-048925-10757
18.6 BARC-018911-03277
40.7 BARC-035255-07160
56.0 BARC-010977-00811
68.5 BARC-057979-15045
76.1 BARC-040749-07839
81.6 BARC-060257-16508
114.1 BARC-015925-02017
0.0 BARC-018693-02992
21.9 BARC-052547-11497
Chromosome 11
0.0 BE806308
18.2 Sat_272
37.2 Satt426
37.8 Sat_411
49.5 BARC-040851-07854
63.3 BARC-042473-08271
70.2 BARC-016279-02316
0.0 BARC-035379-07181
22.2 BARC-900336-00920
28.1 BARC-025703-04996
Chromosome 12
0.0 BARC-032147-07327
5.1 BARC-062675-18002
16.6 BARC-056033-13997
17.3 BARC-061985-17608
23.2 BARC-018895-03034
32.6 BARC-044073-08598
40.8 BARC-049209-10821
46.9 BARC-032647-09003
60.4 BARC-039237-07479

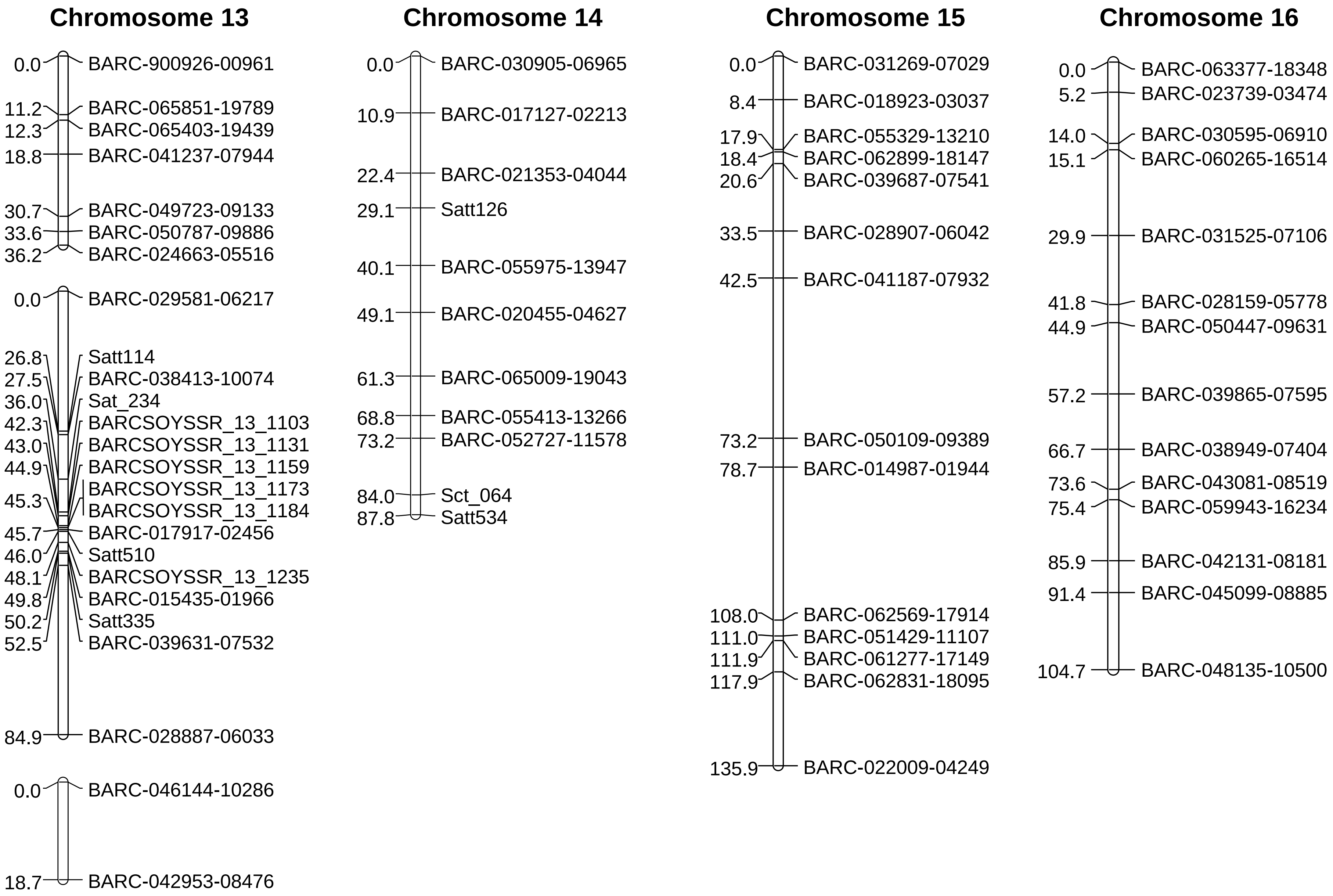
Chromosome 13
0.0 BARC-900926-00961
11.2 BARC-065851-19789
12.3 BARC-065403-19439
18.8 BARC-041237-07944
30.7 BARC-049723-09133
33.6 BARC-050787-09886
36.2 BARC-024663-05516
0.0 BARC-029581-06217
26.8 Satt114
27.5 BARC-038413-10074
36.0 Sat_234
42.3 BARCSOYSSR_13_1103
43.0 BARCSOYSSR_13_1131
44.9 BARCSOYSSR_13_1159
45.3 BARCSOYSSR_13_1173
BARCSOYSSR_13_1184
45.7 BARC-017917-02456
46.0 Satt510
48.1 BARCSOYSSR_13_1235
49.8 BARC-015435-01966
50.2 Satt335
52.5 BARC-039631-07532
84.9 BARC-028887-06033
0.0 BARC-046144-10286
18.7 BARC-042953-08476
Chromosome 14
0.0 BARC-030905-06965
10.9 BARC-017127-02213
22.4 BARC-021353-04044
29.1 Satt126
40.1 BARC-055975-13947
49.1 BARC-020455-04627
61.3 BARC-065009-19043
68.8 BARC-055413-13266
73.2 BARC-052727-11578
84.0 Sct_064
87.8 Satt534
Chromosome 15
0.0 BARC-031269-07029
8.4 BARC-018923-03037
17.9 BARC-055329-13210
18.4 BARC-062899-18147
20.6 BARC-039687-07541
33.5 BARC-028907-06042
42.5 BARC-041187-07932
73.2 BARC-050109-09389
78.7 BARC-014987-01944
108.0 BARC-062569-17914
111.0 BARC-051429-11107
111.9 BARC-061277-17149
117.9 BARC-062831-18095
135.9 BARC-022009-04249
Chromosome 16
0.0 BARC-063377-18348
5.2 BARC-023739-03474
14.0 BARC-030595-06910
15.1 BARC-060265-16514
29.9 BARC-031525-07106
41.8 BARC-028159-05778
44.9 BARC-050447-09631
57.2 BARC-039865-07595
66.7 BARC-038949-07404
73.6 BARC-043081-08519
75.4 BARC-059943-16234
85.9 BARC-042131-08181
91.4 BARC-045099-08885
104.7 BARC-048135-10500

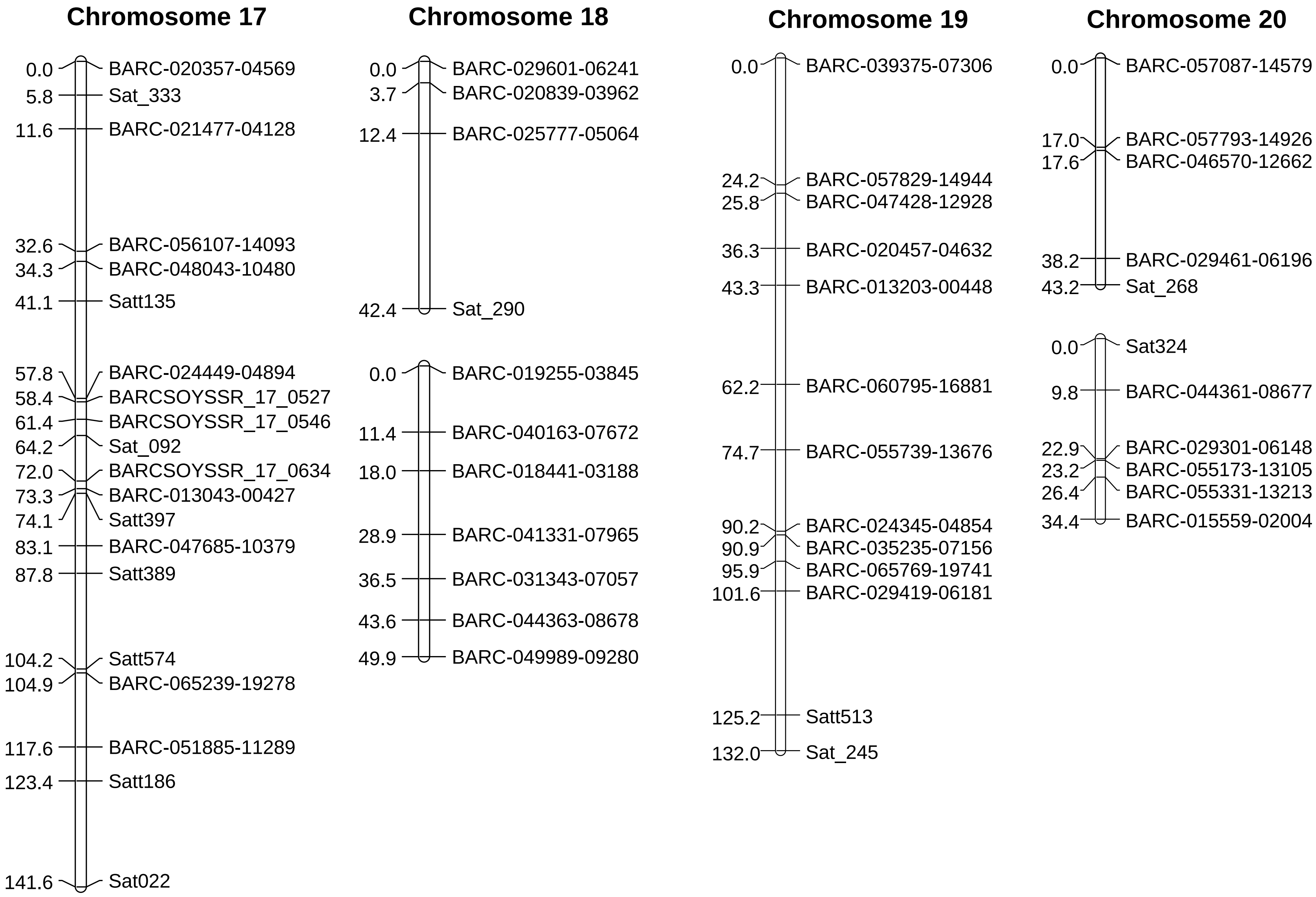
Chromosome 17
0.0 BARC-020357-04569
5.8 Sat_333
11.6 BARC-021477-04128
32.6 BARC-056107-14093
34.3 BARC-048043-10480
41.1 Satt135
57.8 BARC-024449-04894
58.4 BARCSOYSSR_17_0527
61.4 BARCSOYSSR_17_0546
64.2 Sat_092
72.0 BARCSOYSSR_17_0634
73.3 BARC-013043-00427
74.1 Satt397
83.1 BARC-047685-10379
87.8 Satt389
104.2 Satt574
104.9 BARC-065239-19278
117.6 BARC-051885-11289
123.4 Satt186
141.6 Sat022
Chromosome 18
0.0 BARC-029601-06241
3.7 BARC-020839-03962
12.4 BARC-025777-05064
42.4 Sat_290
0.0 BARC-019255-03845
11.4 BARC-040163-07672
18.0 BARC-018441-03188
28.9 BARC-041331-07965
36.5 BARC-031343-07057
43.6 BARC-044363-08678
49.9 BARC-049989-09280
Chromosome 19
0.0 BARC-039375-07306
24.2 BARC-057829-14944
25.8 BARC-047428-12928
36.3 BARC-020457-04632
43.3 BARC-013203-00448
62.2 BARC-060795-16881
74.7 BARC-055739-13676
90.2 BARC-024345-04854
90.9 BARC-035235-07156
95.9 BARC-065769-19741
101.6 BARC-029419-06181
125.2 Satt513
132.0 Sat_245
Chromosome 20
0.0 BARC-057087-14579
17.0 BARC-057793-14926
17.6 BARC-046570-12662
38.2 BARC-029461-06196
43.2 Sat_268
0.0 Sat324
9.8 BARC-044361-08677
22.9 BARC-029301-06148
23.2 BARC-055173-13105
26.4 BARC-055331-13213
34.4 BARC-015559-02004
