# Supplementary material for: Novel quantitative trait loci for partial resistance to Phytophthora sojae in soybean PI 398841
Source: Theor Appl Genet. 2013 Jan 25;126(4):1121–32. doi: 10.1007/s00122-013-2040-x (PMC3607739; doi:10.1007/s00122-013-2040-x)
Supplement: Supplementary file 2 — Supplementary material 2 The response of OX20-8, PI 398841, and fifteen differentials following inoculations with forty-eight isolates of Phytophthora sojae (DOCX 29 kb) [file 122_2013_2040_MOESM2_ESM.docx]

**Supplementary Table 1** Interaction between *Rps* genes in OX20-8, PI 398841, and fifteen differentials and *Avr* genes in 48 isolates of *P. sojae*

| Genotype | *Rps* gene | *Phytophthora sojae* isolates (pathotypes) ^a^ | | | | | | | | | | | | | | | |
| --- | --- | --- | --- | --- | --- | --- | --- | --- | --- | --- | --- | --- | --- | --- | --- | --- | --- |
|  |  | OH 1 | OH 2 | OH 3 | OH 4 | OH 17 | OH 25 | C2S1 | 1.S.1.1 | 2010 DF 1-1 | 2010 DF 1-1 (2) | 2010 DF 2(2)4 | 2010 DF 3-1 | 2010 DF 3-5 | 2010 DF 9 | 2010 DP 4 | 2010 DP 5 |
| OX20-8 | *Rps1a* | R | S | S | S | R | S | S | S | S | S | S | S | S | S | S | S |
| PI 398841 | Unknown | S | S | S | S | S | S | S | S | S | I | S | S | S | S | S | S |
| Williams | *rps* | S | S | S | S | S | S | S | S | S | S | S | S | S | S | S | I |
| Harlon | *Rps1a* | R | I | S | S | R | S | S | S | S | S | I | S | S | S | S | R |
| Harosoy 13XX | *Rps1b* | R | S | R | R | S | S | S | S | S | I | S | S | S | - | S | R |
| Williams79 | *Rps1c* | R | R | R | S | R | S | S | R | S | R | S | I | S | - | I | R |
| PI 103091 | *Rps1d* | I | R | R | I | S | R | I | I | S | I | I | I | S | I | S | R |
| Williams82 | *Rps1k* | R | R | R | R | R | S | S | S | S | S | S | S | S | S | S | R |
| L76-1988 | *Rps2* | R | R | R | R | S | R | S | S | S | R | S | R | S | R | S | I |
| L83-570 | *Rps3a* | R | R | R | I | S | R | S | S | S | R | S | R | I | I | I | I |
| PRX-146-36 | *Rps3b* | R | R | R | R | S | R | S | S | I | R | R | R | I | R | I | R |
| PRX-145-48 | *Rps3c* | R | R | R | R | S | R | S | S | I | R | I | I | I | R | R | R |
| L85-2352 | *Rps4* | R | R | R | R | S | R | S | S | I | R | I | R | I | R | I | R |
| L85-3059 | *Rps5* | R | R | R | R | S | R | S | S | I | R | I | R | S | R | I | I |
| Harosoy 62XX | *Rps6* | R | R | R | R | S | R | S | S | R | R | S | R | I | R | I | R |
| Harosoy | *Rps7* | S | S | S | S | S | S | S | S | S | S | S | S | S | S | R | S |
| PI 399073 | *Rps8* | R | - | R | R | S | R | R | S | I | R | S | I | S | R | I | I |

^a^ R, I, and S denote the genotypes were resistant, intermediate, or susceptible to the isolate and missing results were indicated by a hyphen

**Supplementary Table 1** (Cont’d)

| Genotype | *Rps* gene | *Phytophthora sojae* isolates (pathotypes) ^a^ | | | | | | | | | | | | | | | |
| --- | --- | --- | --- | --- | --- | --- | --- | --- | --- | --- | --- | --- | --- | --- | --- | --- | --- |
|  |  | 2010 DP 7 | 2010 DP 8 | 2010 DP 9 | 2010 DPH 3 | 2010 DPH 4 | 2010 WI 2 | 2010 WI 3 | Ash 1-6-13 S.1 | OH 1999 76.5.3.1 S.1 | OH 2000 Sandusky 1 S.1 | OH 2000 Sandusky 6 S.2 | OH 2000 Sandusky 38 S.1 | OH 2000 Sandusky 72 S.1 | OH 2000 Sandusky 84 S.1 | OH 2000 Wood 1 S.1 | OH 2000 Wood 58 S.1 |
| OX20-8 | *Rps1a* | S | S | R | S | I | I | S | S | S | S | S | S | S | S | S | S |
| PI 398841 | Unknown | S | S | S | S | I | I | S | S | S | S | S | S | S | S | I | S |
| Williams | *rps* | S | S | S | S | S | I | S | S | S | S | S | S | S | S | S | S |
| Harlon | *Rps1a* | S | S | I | I | I | I | S | S | S | S | S | R | S | S | S | S |
| Harosoy 13XX | *Rps1b* | R | S | I | S | R | I | S | S | R | I | I | R | S | R | S | S |
| Williams79 | *Rps1c* | S | I | I | R | S | S | S | S | R | S | S | R | S | S | S | I |
| PI 103091 | *Rps1d* | I | I | I | I | I | S | S | S | I | I | S | I | S | I | R | S |
| Williams82 | *Rps1k* | R | S | S | S | R | S | S | S | I | I | I | R | S | R | S | S |
| L76-1988 | *Rps2* | S | S | R | R | S | R | R | I | I | S | S | I | I | R | R | S |
| L83-570 | *Rps3a* | S | I | R | R | R | R | I | S | R | S | S | R | R | I | R | S |
| PRX-146-36 | *Rps3b* | R | I | R | R | R | R | R | S | R | S | I | R | S | R | R | I |
| PRX-145-48 | *Rps3c* | I | R | R | R | R | R | R | I | R | I | I | R | R | R | R | R |
| L85-2352 | *Rps4* | R | I | R | R | R | I | R | R | R | R | R | R | R | R | R | R |
| L85-3059 | *Rps5* | I | S | R | I | I | R | S | I | R | S | I | I | I | I | I | S |
| Harosoy 62XX | *Rps6* | R | I | R | R | R | R | R | R | R | R | R | R | R | R | R | R |
| Harosoy | *Rps7* | S | S | R | S | S | S | S | S | S | S | S | S | S | S | S | S |
| PI 399073 | *Rps8* | S | S | R | S | R | R | I | I | R | S | I | I | R | I | R | R |

^a^ R, I, and S denote the genotypes were resistant, intermediate, or susceptible to the isolate and missing results were indicated by a hyphen

**Supplementary Table 1**(Cont’d)

| Genotype | *Rps* gene | *Phytophthora sojae* isolates (pathotypes) ^a^ | | | | | | | | | | | | | | | |
| --- | --- | --- | --- | --- | --- | --- | --- | --- | --- | --- | --- | --- | --- | --- | --- | --- | --- |
|  |  | OH 2000 Wood 69 S.1 | OH 2000 Wood 80 S.1 | OH SS03 | OH SS03 Fulton 3 | OH SS03 Henry Co | OH SS03 Resnik 3 | OH SS03 Wayne 5 S.1 | OH SS03 Wood Co 5 S.1 | OH SS04 Defiance Co 309-15-1 S.1 | OH SS04 Holmes As 2705 | OH SS04 Miami Co 1 | OH SS04 NWB S.1 | OH SS05 Butler Co S.1 | OH SS05 Delaware 1 | OH SS05 Paulding Co 3A-1 | OH SS07 Fair field 6 S.1 |
| OX20-8 | *Rps1a* | S | S | S | S | S | S | S | S | S | S | S | S | S | I | S | S |
| PI 398841 | Unknown | S | S | S | S | S | I | S | S | S | I | S | S | S | S | S | S |
| Williams | *rps* | S | S | S | S | S | I | S | S | S | S | S | S | S | S | S | S |
| Harlon | *Rps1a* | S | S | S | S | S | I | S | S | S | S | S | S | S | S | S | S |
| Harosoy 13XX | *Rps1b* | S | I | I | S | I | I | S | S | S | S | S | I | S | I | S | S |
| Williams79 | *Rps1c* | S | S | S | R | R | I | I | I | S | S | I | I | S | R | I | S |
| PI 103091 | *Rps1d* | S | R | S | I | S | R | S | I | S | I | S | S | S | S | S | I |
| Williams82 | *Rps1k* | S | S | I | S | R | R | S | S | S | S | S | R | S | R | S | S |
| L76-1988 | *Rps2* | S | I | I | S | S | I | I | S | S | R | I | S | S | S | S | S |
| L83-570 | *Rps3a* | S | R | I | I | S | R | S | S | S | R | S | S | S | S | S | R |
| PRX-146-36 | *Rps3b* | S | R | I | R | R | R | I | S | S | R | I | S | S | S | I | R |
| PRX-145-48 | *Rps3c* | I | R | R | R | S | R | R | I | R | R | R | S | S | S | R | R |
| L85-2352 | *Rps4* | S | R | I | R | S | R | S | R | R | R | R | S | R | S | R | R |
| L85-3059 | *Rps5* | S | R | S | S | S | R | R | S | S | I | S | S | S | S | I | S |
| Harosoy 62XX | *Rps6* | R | R | R | R | S | R | R | R | R | R | R | I | R | S | R | R |
| Harosoy | *Rps7* | S | S | S | S | S | S | S | S | S | S | S | S | S | S | S | S |
| PI 399073 | *Rps8* | S | R | R | R | I | R | R | S | I | R | I | S | S | S | S | R |

^a^ R, I, and S denote the genotypes were resistant, intermediate, or susceptible to the isolate and missing results were indicated by a hyphen
